# *In vitro* iron nanoparticles modulate ginsenoside biosynthesis and trigger ROS-mediated stress response in ginseng adventitious roots

**DOI:** 10.1016/j.jgr.2026.101118

**Published:** 2026-08-06

**Authors:** Unenzaya Bayaraa, Jin Hoon Jang, Sudheeran Pradeep Kumar, Ok Ran Lee

**Affiliations:** a Department of Applied Plant Science, College of Agriculture and Life Science, Chonnam National University, Gwangju, 61186, Republic of Korea; b Interdisciplinary Program in IT-Bio Convergence System, Chonnam National University, Gwangju, 61186, Republic of Korea; c Laboratory of Plant Biotechnology, Institute of Biology, Mongolian Academy of Sciences, Ulaanbaatar, 13330, Mongolia

**Keywords:** Ginseng adventitious roots, Ginsenoside biosynthesis, Iron nanoparticles, ROS, Elicitation

## Abstract

**Background:**

Enhancing ginsenoside accumulation is essential for improving the medicinal quality of *Panax ginseng*. Although iron nanoparticles (Fe-NPs) have emerged as promising elicitors of secondary metabolism, their specific effects on ginsenoside biosynthesis remains elusive.

**Methods:**

Ginseng adventitious roots were treated with Fe-NP treatment in comparison with a conventional iron source (Fe_3_O_4_). Physiological responses, ginsenoside profiles, and the expression of key biosynthetic and reactive oxygen species (ROS)-related genes were analyzed to compare elicitation efficiency and potential toxicity.

**Results:**

At a lower concentration (10 ϻM), Fe-NP concentrations markedly induced key ginsenoside-biosynthetic genes and increased total ginsenoside content by 14-16%, whereas equivalent concentrations of Fe_3_O_4_ caused no significant metabolic changes, demonstrating the superior elicitation efficiency of the nanoparticle formulation. In contrast, higher Fe-NP doses (50-100 μM) caused iron toxicity and suppressed ginsenoside accumulation, likely through excessive ROS generation as supported by strong induction of ROS-detoxification genes. Notably, ginsenoside Re levels increased under iron stress, suggesting a potential antioxidative role.

**Conclusion:**

Overall, our findings reveal a dose-dependent dual effect of Fe-NPs, promoting ginsenoside biosynthesis at optimal doses while triggering ROS-mediated suppression at higher levels, and highlight the importance of precise Fe-NP dosage optimization for biotechnological applications.

## Graphical abstract

**Figure:**
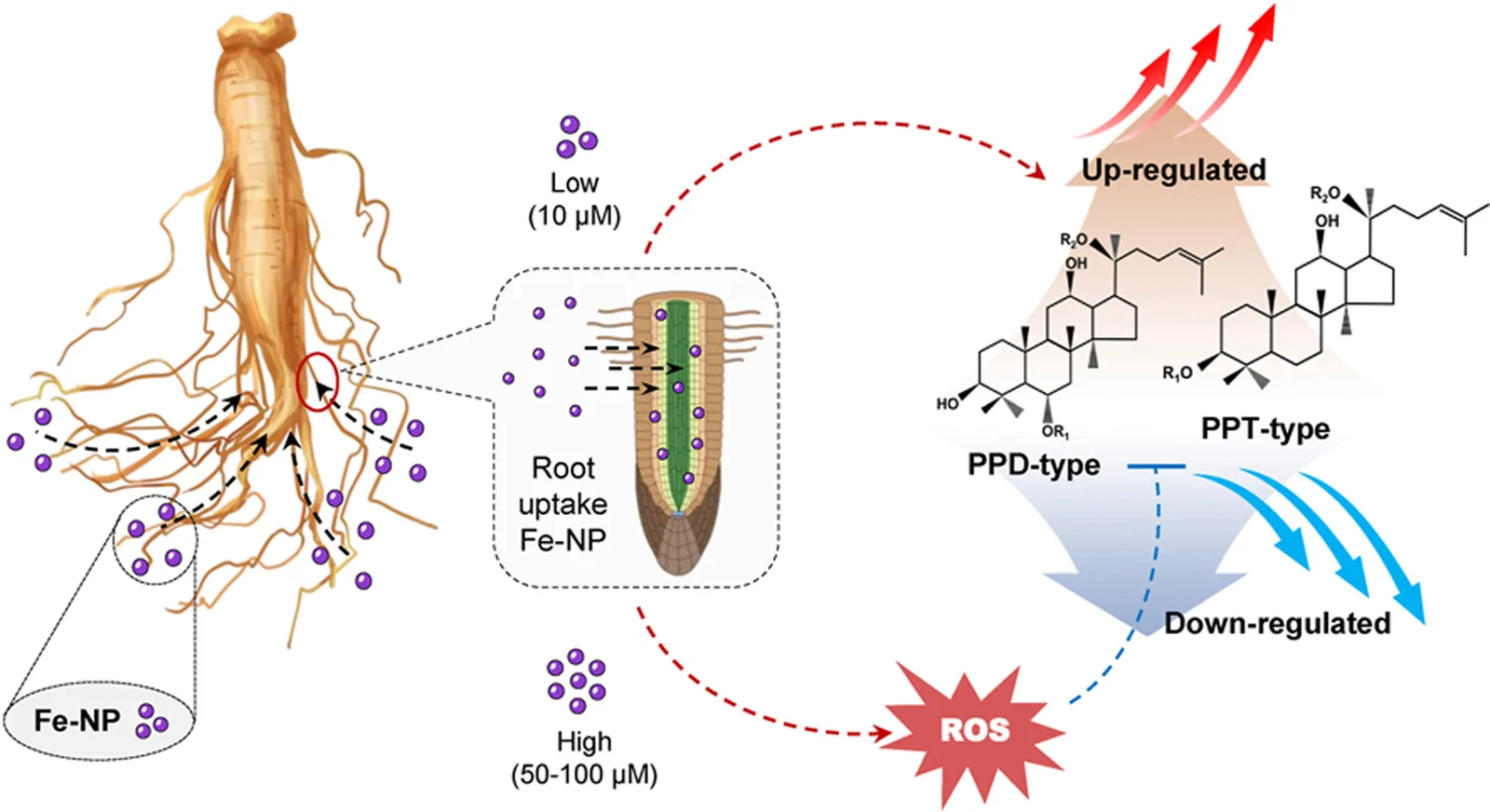

## 1 Introduction

*Panax ginseng* Meyer (Korean ginseng), has long been valued in traditional medicine, particularly across East Asia. Its pharmacological significance is attributed to a rich repertoire of triterpenoid saponins, collectively termed ginsenosides, which exert a broad spectrum of beneficial effects on human health, including improvements in cognitive performance and physiological resilience [1]. Structurally, ginsenosides are categorized into two principal classes: oleanane- and dammarane-type saponins, which are distinguished by differences in their aglycone cores and the arrangement of sugar residues [2]. Dammarane-type saponins are generally classified into three major subclasses, protopanaxadiol (PPD), protopanaxatriol (PPT), and ocotillol. Each group exhibits distinct bioactivities, including neuroprotective, anti-cancer, anti-diabetic, antioxidant, and anti-inflammatory effects, underscoring their therapeutic relevance [1,3].

In *P. ginseng*, the ginsenoside biosynthesis pathway is initiated from the mevalonate (MVA) pathway, with 3-hydroxy-3-methylglutaryl-CoA reductase (*PgHMGR1***)** catalyzing the rate-limiting conversion of HMG-CoA to mevalonate [2], thereby controlling precursor supply for downstream terpenoid synthesis. Squalene epoxidase (*PgSQE1*) subsequently catalyzes squalene oxidation to 2,3-oxidosqualene, a pivotal branch point for triterpene formation [4,5]. Oxidosqualene is then cyclized by dammarenediol-II synthase (*PgDDS*) to form the dammarane core of major ginsenosides, highlighting its key regulatory role in the pathway [6]. Beyond these core enzymes, specific cytochrome P450s, including oleanolic acid synthase (*PgOAS*), protopanaxadiol synthase (*PgPPDS*), and protopanaxatriol synthase (*PgPPTS*), catalyze formation of distinct aglycone backbones essential for oleanane, protopanaxadiol, and protopanaxatriol type ginsenosides, respectively, contributing to the structural diversity of ginsenosides in ginseng [1].

Ginseng has attracted increasing interest for its medicinal value, largely driven by the demand for its saponins, which display a broad spectrum of therapeutic activities [7]. However, ginseng's slow growth, limited seed yield, and extended generation time have led to a long-standing gap between ginsenoside demand and availability [8]. To address this gap, techniques like artificial ginsenoside synthesis using yeast-derived precursors and the creation of transgenic plants have been explored [9,10]. Despite their potential, these methods are constrained by high production costs, labor-intensive processes, and long timelines for developing viable crops through plant transformation. Currently, elicitation is recognized as an effective strategy to enhance the biosynthesis of plant-derived specialized metabolites, offering higher productivity while lowering production costs [[11], [12], [13], [14]].

Micronutrients including iron (Fe), zinc (Zn), copper (Cu), and manganese (Mn) are indispensable for plant development, where they participate in a wide range of physiological functions and metabolic pathways [[15], [16], [17]]. Beyond their critical roles, these micronutrients also act as signaling molecules, influencing physiological and biochemical processes that regulate plant responses to environmental stimuli [18]. The introduction of these micronutrients in nanoparticle (NP) form offers several benefits to agriculture by improving nutrient uptake efficiency due to the small particle size (1 nm to 100 nm) [19,20]. Among various NP-based micronutrients, iron is crucial for photosynthesis and enzymatic activities in plants [21]. Previous studies suggest that iron nanoparticles (Fe-NP) have better bioavailability than conventional iron forms, making it easier for plants to absorb and utilize this micronutrient [22]. Numerous reports indicate that external supplementation with Fe-NP can significantly stimulate the accumulation of bioactive metabolites such as flavonoids and carotenoids [[23], [24], [25]], making Fe-NP as promising agent for enhancing plant quality from the agricultural technology perspective.

Enhancing ginsenoside content in ginseng roots via Fe-NP elicitation has the potential to boost agricultural productivity and benefit the pharmaceutical industry. However, the effects of Fe-NP exposure on ginseng adventitious roots and ginsenoside accumulation remain poorly understood. Therefore, this study aimed to evaluate the impact of Fe-NPs on ginsenoside production in ginseng adventitious roots. As a treatment, the adventitious roots of ginseng were subjected to various levels of Fe-NP (0, 10, 50 and 100 μM) for 96 h. After treatment, ginsenoside content, root biomass, target genes expression, and ROS levels were measured to elucidate how Fe-NP influences ginsenoside biosynthesis.

## 2 Materials and methods

### 2.1 Plant materials, culture and growth conditions

*P*. *ginseng* adventitious root (cv. Chunpoong) was generated from the embryonic callus based on previously established method [26]. In brief, the seeds were germinated through water spraying and stratification at 4°C in dark conditions. Seeds with the testa removed were first disinfected by immersion in 70% ethanol for 1 min, followed by treatment with 2% sodium hypochlorite supplemented with 0.05% Tween 20 for 20 min, and subsequently washed several times with sterile distilled water. Following surface sterilization, embryos were isolated and placed on MS-based callus induction medium (3% sucrose, 1 mg/L 2,4-D, 0.5 mg/L 6-BAP, 0.8% phytoagar; pH 5.7) and maintained in the dark at 23°C. The resulting calli were transferred to B5 medium (3% sucrose, 3 mg/L IBA, 0.8% phytoagar; pH 5.7) to promote adventitious root formation, also under dark conditions at 23°C. For mass propagation, adventitious roots were cultured in B5 liquid medium supplemented with 3 mg/L IBA for 21 days in darkness (Fig. S1).

### 2.2 Iron oxide nanoparticle, conventional iron and treatment condition

The Fe-NP (Fe_3_O_4_-NP) were purchased from Houston, USA (product number: US3220, >98% purity), The aerodynamic) particles exhibited a size of 20-30 nm diameter, a specific surface area of 40-60 m^2^/g, and a spherical morphology. The bulk density was 0.84 g/cm^3^, while the true density ranged from 4.8 to 5.1 g/cm^3^. A 1 M stock solution of Fe-NP was prepared by dissolving it into double-distilled water, vortexing for approximately 5 min, and then incubated at 60°C for 30 min and then autoclaving the solution. After a 21-day period in the liquid medium, adventitious roots were treated with Fe-NP, whereas adventitious roots cultured for 28 days were treated with a non-nanoparticle iron source, Fe_3_O_4_. Both treatments were appliedat concentrations of 0, 10, 50, and 100 ϻM, respectively, in a freshly prepared B5 liquid medium for 96 h (Fig. S1). This treatment was conducted with three biological replicates, with each replicate consisting of 24 ginseng adventitious roots. Following the 96h treatment, the adventitious roots were collected, dried in filter paper, and their biomass was measured. Samples were rapidly frozen and preserved at −80 °C for later examination.

### 2.3 Transmission electron microscopy (TEM) and energy-dispersive x-ray spectroscopy (EDS)

Samples were fixed in 0.05 M cacodylate buffer (pH 7.2) containing 2% glutaraldehyde and 2% paraformaldehyde, dehydrated in graded ethanol (30-100%), and embedded in LR white resin. Following polymerization at 60 °C for 24 h, ultrathin sections (80-100 nm) were prepared, stained with uranyl acetate and lead citrate, and examined by TEM (JEM-ARM200F NEO ARM; JEOL, Japan). Elemental composition was determined by EDS (JEOL dual-detector system), and spectra were processed using AZtecTEM software (Oxford Instruments, UK).

### 2.4 cDNA synthesis and quantification of gene expression

Freshly ground adventitious roots were subjected to RNA extraction using the TaKaRa MiniBEST Plant RNA Extraction Kit (Takara, Japan). A total of 4 ϻg of RNA was reverse transcribed into cDNA with the RevertAid cDNA Synthesis Kit (Thermo, USA). Quantitative PCR (qPCR) was carried out using TB Green™ Premix Ex Taq™ on a Thermal Cycler Dice system (Takara, Japan), with gene expression levels normalized to *β-actin* and calculated according to the 2^−ΔΔCt^ method. Primer sequences used in this study are listed in Table S1.

### 2.5 Ginsenoside profiling and quantification

Methanolic extracts of 200 mg lyophilized ginseng adventitious root powder were subjected to sonication at 40°C for 30 min, followed by centrifugation at 3000 ✕ g for 10 min, and filtered through a SepPak C18 cartridge. Chromatographic separation was conducted on a YMC-Pack Pro C18 RS column (150 ✕ 2.0 mm, 5 μm) maintained at 40°C, employing a water-acetonitrile gradient at a flow rate of 0.5 mL/min. Detection was performed using a photodiode array at 200 nm, coupled to an LC/MS-IT-TOF system (Shimadzu, Japan) equipped with APCI in both positive and negative ionization modes. The instrument was operated with a detector voltage of 1.60 kV, N_2_ nebulizing gas at 1.5 L/min, TOF pressure of 1.5 ✕ 10^-4^ Pa, ion trap pressure of 1.7 ✕ 10^-2^ Pa, 30 ms ion accumulation, a 3.0 amu precursor width, and MS1/MS2 scan ranges of 0-1000 and 100-500 *m*/*z*, respectively.

### 2.6 In situ H_2_O_2_ detection by DAB staining and colorimetric assay

H_2_O_2_ accumulation in adventitious roots treated with 0, 10, 50, and 100 μM Fe-NP was observed *in situ* through 3,3′-diaminobenzidine (DAB) staining, which forms brown precipitates upon reacting with H_2_O_2_. Roots were incubated in 1 mg/mL DAB solution containing 200 mM Na_2_HPO_4_ and 0.05% Tween 20, subjected to 10 min vacuum infiltration, and incubated at 25°C for 4 h with shaking at 80 rpm. Samples were then bleached in a boiling ethanol-acetic acid-glycerol (3:1:1) mixture at 90°C for 15 min, transferred to 70% ethanol, and imaged under a light microscope (DM3000 LED, Leica, Germany). H_2_O_2_ levels were quantified using an H_2_O_2_ assay kit (BO-PER500, BIOMAX, Seoul, Korea) per the manufacturer's protocol.

### 2.7 Statistical methods

Data is presented as mean ± SD. One-way ANOVA with Tukey's post hoc test was used to evaluate statistical significance at *P < *0.05, using SAS version 9.4 (SAS Institute, USA).

## 3 Results

### 3.1 Uptake and intracellular localization of Fe_3_O_4_ nanoparticles in ginseng adventitious roots

To determine whether Fe_3_O_4_ nanoparticles (Fe-NPs) could penetrate ginseng adventitious roots, TEM analysis was performed. Compared with the untreated control, roots exposed to 10, 50, and 100 μM Fe-NPs showed clear nanoparticle accumulation within root cells, confirming successful penetration and uptake (Fig. 1A). Electron-dense nanoparticle aggregates were observed in root tissues, including the central vacuole. EDS mapping further verified the identity of these deposits, showing co-localization of Fe and O signals characteristic of Fe_3_O_4_ nanoparticles (Fig. 1B). These findings demonstrate that Fe_3_O_4_ nanoparticles successfully penetrate and accumulate within ginseng adventitious roots while retaining their original chemical composition.

**Fig. 1:**
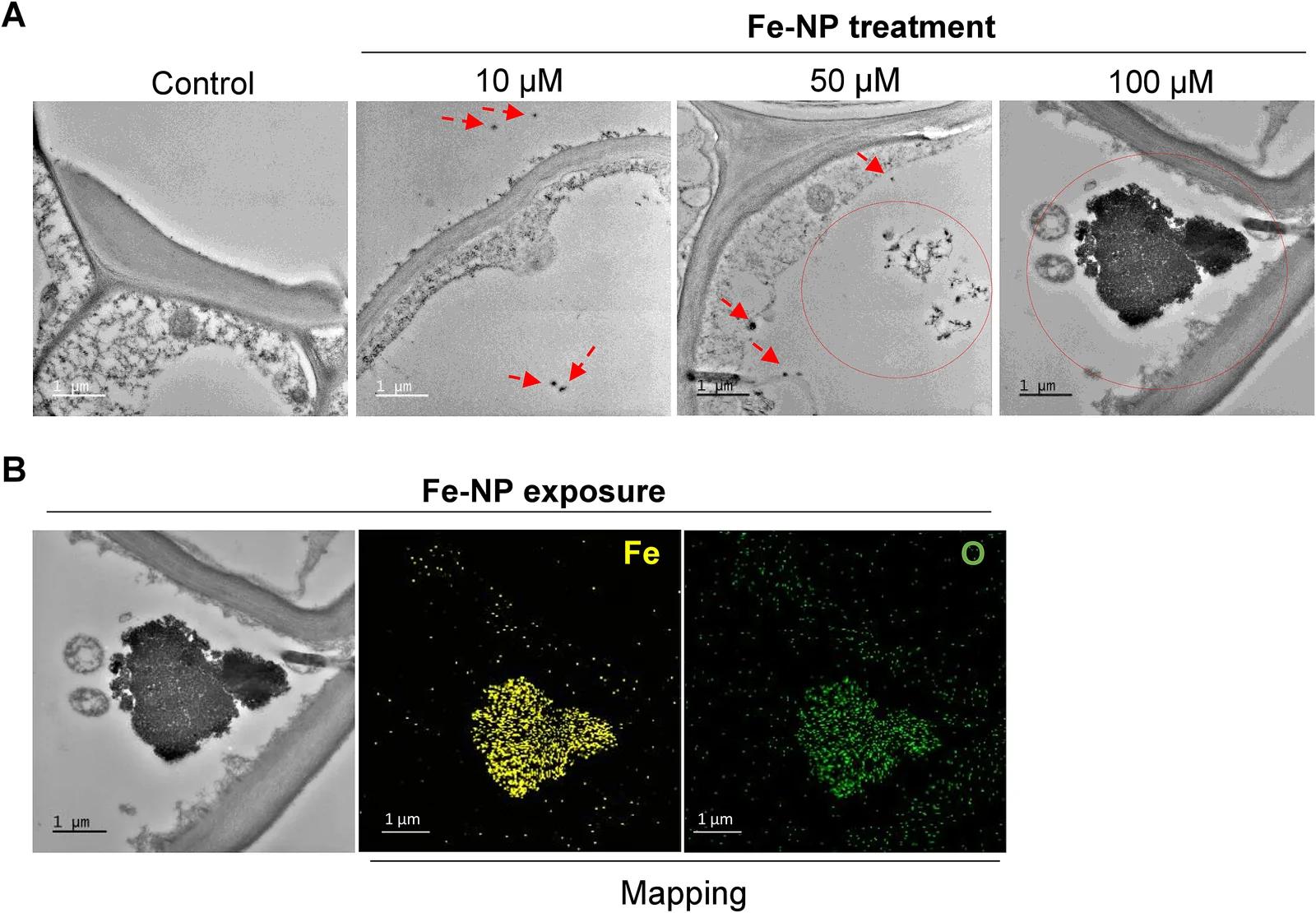
Cellular and elemental localization of Fe_3_O_4_ nanoparticles (Fe-NPs) in ginseng adventitious roots. (A) Transmission electron microscopy (TEM) images showing Fe-NP penetration into root tissue following Fe-NP treatment, compared with untreated control roots. Red arrows indicate electron-dense, scattered nanoparticle aggregates consistent with internalized Fe-NPs, which were absent in control samples. (B) Energy-dispersive X-ray spectroscopy (EDS) elemental mapping confirming the presence of Fe-NPs within Fe-NP-treated ginseng adventitious root tissue. Yellow signals correspond to iron (Fe) and green signals to oxygen (O), with their spatial co-localization consistent with the oxide composition of magnetite (Fe_3_O_4_).

### 3.2 Fe-NP affects the ginsenoside biosynthesis genes

To assess the effects of iron nanoparticles (Fe-NPs) on ginsenoside biosynthesis, ginseng adventitious roots were treated with Fe-NPs (1-100 nm), which are substantially smaller than conventional iron oxide particles (>1 μm) and can therefore more readily enter plant cells [15,20]. We checked the transcript levels of key genes regulating ginsenoside production, *PgHMGR1*, *PgSQE1*, *PgDDS*, *PgOAS*, *PgPPDS*, and *PgPPTS*, under Fe-NP treatment (Fig. 1A). Upon treatment with 10 μM Fe-NP, four of the six tested genes were significantly upregulated (Fig. 2A). In particular, the expression of *PgDDS*, which regulates the dammarane-type ginsenoside backbone, showed a 1.5-fold upregulation (Fig. 2B). Moreover, the expression levels of *PgPPDS, PgPPTS*, and *PgOAS* were increased by approximately 3, 3.5, and 4.3-fold, respectively, suggesting that low concentrations of Fe-NP can serve as potential inducers of dammarane-type ginsenoside biosynthesis. At 50 μM, only *PgPPDS* and *PgPPTS* exhibited increased expression, while the other genes expression was comparable to the control. At 100 μM, *PgOAS* and *PgPPDS* exhibited notable downregulation (Fig. 2B). The reduced expression of ginsenoside biosynthetic genes indicates that Fe-NPs regulate their transcript levels in a negatively correlated, dose-dependent manner.

**Fig. 2:**
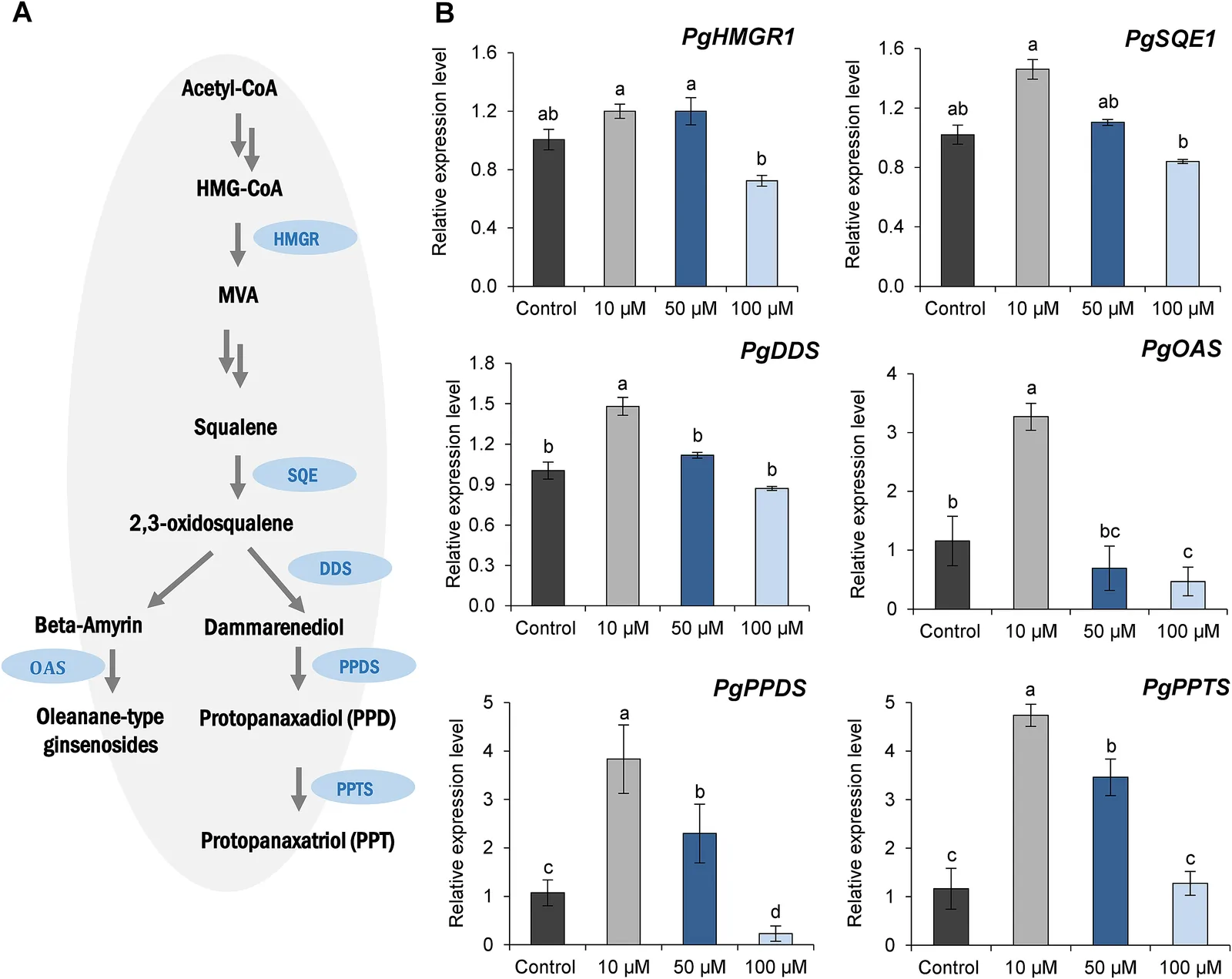
Ginsenoside biosynthesis genes in ginseng adventitious roots under different Fe-NP concentrations. (A) Schematic pathway of the ginsenoside biosynthesis via mevalonate (MVA), including key intermediate metabolites and enzymes involved in each step. Enzymes are shown as blue circles, whereas grey arrows indicate enzymatic steps. (B) The expression of six major genes in the ginsenoside biosynthetic pathway (*PgHMGR1*, *PgSQE1, PgDDS, PgOAS, PgPPDS*, and *PgPPTS*). Data represent means ± SD for three independent replicates (n = 3 per group). Different superscript letters indicate statistically significant differences *(P < *0.05) determined by one-way ANOVA with Tukey's *post hoc* test.

### 3.3 Fe-NP distinctly affects the accumulation of ginsenoside

To test whether Fe-NP-induced changes in ginsenoside biosynthetic gene expression translate into altered ginsenoside yield, we profiled four major ginsenoside groups: PPD, PPT, oleanane, and ocotillol (Fig. 3A, S2, Table S2). Upon Fe-NP treatment, significant changes were observed in PPD and PPT levels, whereas oleanane and ocotillol ginsenosides remained low and relatively stable (Fig. 3A–S2, Table S2). Notably, 10 μM Fe-NP increased total PPT by 17% and total PPD by 10.5% compared with the control, resulting in a 13% increase in total ginsenosides content (Fig. 3A–Table S2). These results indicate that 10 μM Fe-NP positively enhances ginsenoside production. Increasing Fe-NP concentrations produced a contrasting metabolic response, with total ginsenoside content decreasing by up to 7% at 50 μM Fe-NP and declining further by 14.1% at 100 μM (Fig. 3A–Table S2). This reduction was primarily driven by a marked decrease in total PPD levels, which dropped from 5.00 to 3.87 mg/g after exposure to 100 μM Fe-NP. In contrast, total PPT levels were largely unaffected, showing only a slight decrease from 5.60 to 5.53 mg/g (Fig. 3A–Table S2), consistent with the Fe-NP–induced changes in ginsenoside biosynthetic gene expression (Fig. 2B). Nanoparticle conjugation enhances the efficiency of micronutrients by improving their stability, absorption, and cellular delivery [27]. To determine whether the stimulatory effect of Fe-NPs on ginsenoside production was attributable to nanoparticle properties rather than iron oxide itself, ginseng adventitious roots were treated with Fe_3_O_4_ at equivalent concentrations (10, 50, and 100 μM). Unlike Fe-NPs, Fe_3_O_4_ did not enhance total ginsenoside accumulation (Fig. 3B; Table S3). Specifically, at 10 μM conventional iron did not significantly alter ginsenoside content compared with the control. At 50 μM, a reduction in total ginsenosides was observed, driven primarily by decreases in PPD-type ginsenosides, while PPT-type ginsenosides showed a slight increase (Fig. 3B; Table S3). This contrasting response suggests a shift in metabolic flux from the PPD to the PPT branch of the ginsenoside biosynthetic pathway. Overall, Fe_3_O_4_ failed to reproduce the ginsenoside-promoting effect of Fe-NPs and instead altered ginsenoside composition without increasing total yield. These findings indicate that the enhancement of ginsenoside accumulation is a nanoparticle-specific effect rather than a consequence of iron oxide chemistry alone.

**Fig. 3:**
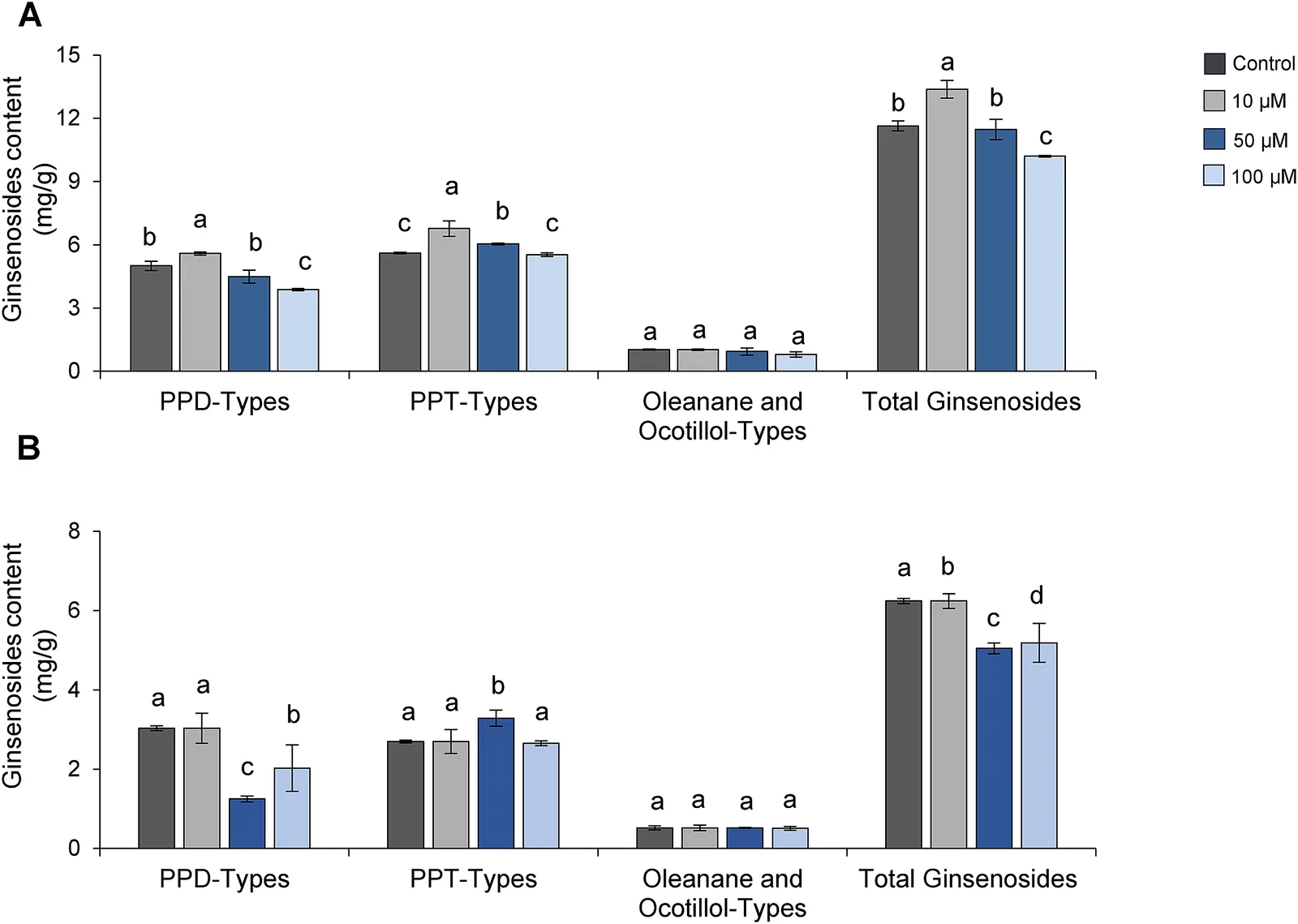
The content of ginsenosides in ginseng adventitious roots under different concentrations of Fe-NP and conventional iron (Fe_3_O_4_). (A) PPD, PPT, oleanane, ocotillol, and total ginsenosides content under various concentrations of Fe-NP. (B) PPD, PPT, oleanane, ocotillol, and total ginsenosides content under 10 μM Fe_3_O_4_. Data represent means ± SD for three independent replicates (n = 3 per group). Different superscript letters indicate statistically significant differences (*P < *0.05) determined by one-way ANOVA with Tukey's *post hoc* test.

### 3.4 Fe-NP alters the proportion ratio of individual PPD and PPT

Total ginsenosides content showed significant changes in response to varying Fe-NPs concentrations, primarily due to alterations in PPD- and PPT-type ginsenosides (Fig. 3A–Table S2). Therefore, we further examined the profiles of individual PPD- and PPT-type ginsenosides affected by Fe-NPs. Within the PPD-type group, seven ginsenosides, Rc, mRb1, Rd, NR4, Ra1, GypXLVI, and GypXVII, were identified in ginseng adventitious roots (Fig. 4A and B). These PPD-type ginsenosides are commonly found in Korean ginseng roots and are known for health benefits in addition to their physiological roles in ginseng [3]. Analysis of ginsenoside profile revealed significant Fe-NP-induced alterations in the accumulation of six PPD-type ginsenosides, including Rc, mRb1, Rd, NR4, GypXLVI, and GypXVII (Fig. 4A and B). Treatment with 10 μM Fe-NP significantly increased the contents of mRB1 (28.8%), Rd (24.3%), and NR4 (12.4%), while Rc showed an approximate 9.4% increased (Fig. 4A and B, and Table S2). However, the levels of all these PPD-type ginsenosides significantly decreased when the Fe-NP concentrations were increased to 50 μM and 100 μM. For example, Rc content decreased from 2.26 mg/g in the control to 1.84 mg/g at 100 μM Fe-NP (Fig. 4A and B, Table S2). Notably, GypXLVI was the most affected among other PPD-type ginsenoside, with its content dramatically decreasing from 0.68 mg/g to 0.25 mg/g, highlighting the negative impact of excessive Fe-NP on PPD-type ginsenosides biosynthesis (Fig. 4B–Table S2). These results indicate that PPD-type ginsenoside biosynthesis is responsive to Fe-NP treatment, where even small changes in Fe-NP concentration can significantly affect the proportion of individual PPD-type ginsenosides.

**Fig. 4:**
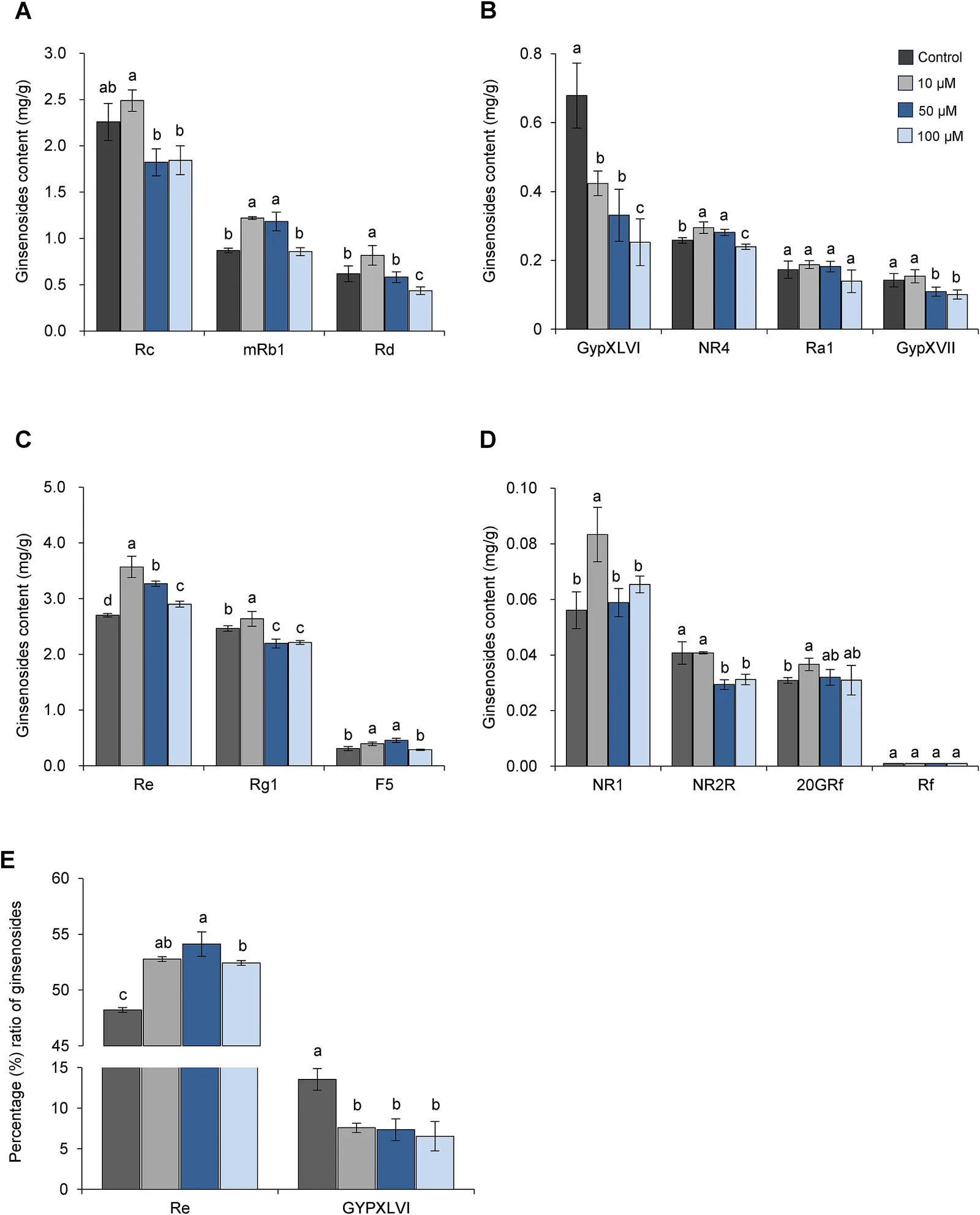
Profiles of PPD and PPT-type ginsenosides in ginseng adventitious roots under different Fe-NP concentrations. (A & B) PPD-type ginsenosides, (C & D) PPT-type ginsenosides, (E) The percentage ratio of Re and GypXLVI upon Fe-NP treatment. Data represent means ± SD for three independent replicates (n = 3 per group). Different superscript letters indicate statistically significant differences (*P < *0.05) determined by one-way ANOVA with Tukey's *post hoc* test.

Changes in PPD-type ginsenosides profiles following Fe-NP treatment were also reflected in their downstream biosynthetic products, the PPT-type ginsenosides. Chromatography analysis detected seven PPT-type ginsenosides, Re, Rg1, F5, NR1, NR2, 20GRf, and Rf, in ginseng adventitious roots (Fig. 4C and D). Furthermore, treatment with 10 μM Fe-NP not only enhanced PPD-type ginsenosides but also promoted a consistent increase in most PPT-type ginsenosides. Among these, Re, Rg1, F5, and NR1, were elevated relatively to the control by up to 24.2%, 6.5%, 21.2%, and 32.6%, respectively (Fig. 4C and D, Table S2). Interestingly, a clear discrepancy emerged between PPD- and PPT-type ginsenosides at higher concentrations of Fe-NP (50 and 100 μM). Unlike the PPD-type, four of seven individual PPT-type ginsenosides, Re, F5, NR1, and 20GRf, were largely unaffected by increased Fe-NP concentration (Fig. 4C and D, Table S2). Notably, among the other PPT-types ginsenosides, Re, the most abundant compound, remained approximately 5% higher (2.90 mg/g) than the control (2.70 mg/g) even at 100 μM Fe-NP. This suggests that ginseng adventitious roots preferentially maintain Re and other PPT-type ginsenosides under high Fe-NP concentration (Fig. 4C–Table S2). Based on these findings, we calculated the percentage contribution of individual ginsenosides at each Fe-NP concentration by normalizing their levels to the total PPD- or PPT-type ginsenosides (Fig. S3). Interestingly, GypXLVI showed the largest decrease in relative abundance among PPD-type ginsenosides, declining from 13.6% to 6.5%, whereas the proportion of Re increased markedly from 48.2% to 52.4% at 100 μM Fe-NP (Fig. 4E–Table S2). These findings indicate that high Fe-NP concentrations suppress ginsenoside biosynthesis primarily by reducing the relative contribution of most PPD-type ginsenosides while favoring the accumulation of PPT-type ginsenosides, particularly Re.

### 3.5 Fe-NP trigger ROS production

The ginsenoside profiles displayed dynamic changes in response to varying concentrations of Fe-NP. Notably, most ginsenosides significantly decreased at high Fe-NP levels, with a relative enrichment of Re ginsenoside (Fig. 4). Previous studies have shown that excessive accumulation of metal NPs can induce abiotic stress, leading to increased ROS production [28]. To assess the potential effects of high Fe-NP concentrations on ROS accumulation, H_2_O_2_ levels in the ginseng adventitious roots were examined using the DAB histochemical staining and colorimetric quantification assay. The staining intensity increased progressively with rising Fe-NP concentrations, particularly at the lateral tips (Fig. 5A). Consistent with these observations, H_2_O_2_ levels increased by 1.6-, 6.8-, and 10.7-fold relative to the control in the 10, 50, and 100 μM Fe-NP treatments, respectively (Fig. 5B). These results indicate that Fe-NP triggers ROS accumulation in ginseng adventitious roots in a dose-dependent manner.

**Fig. 5:**
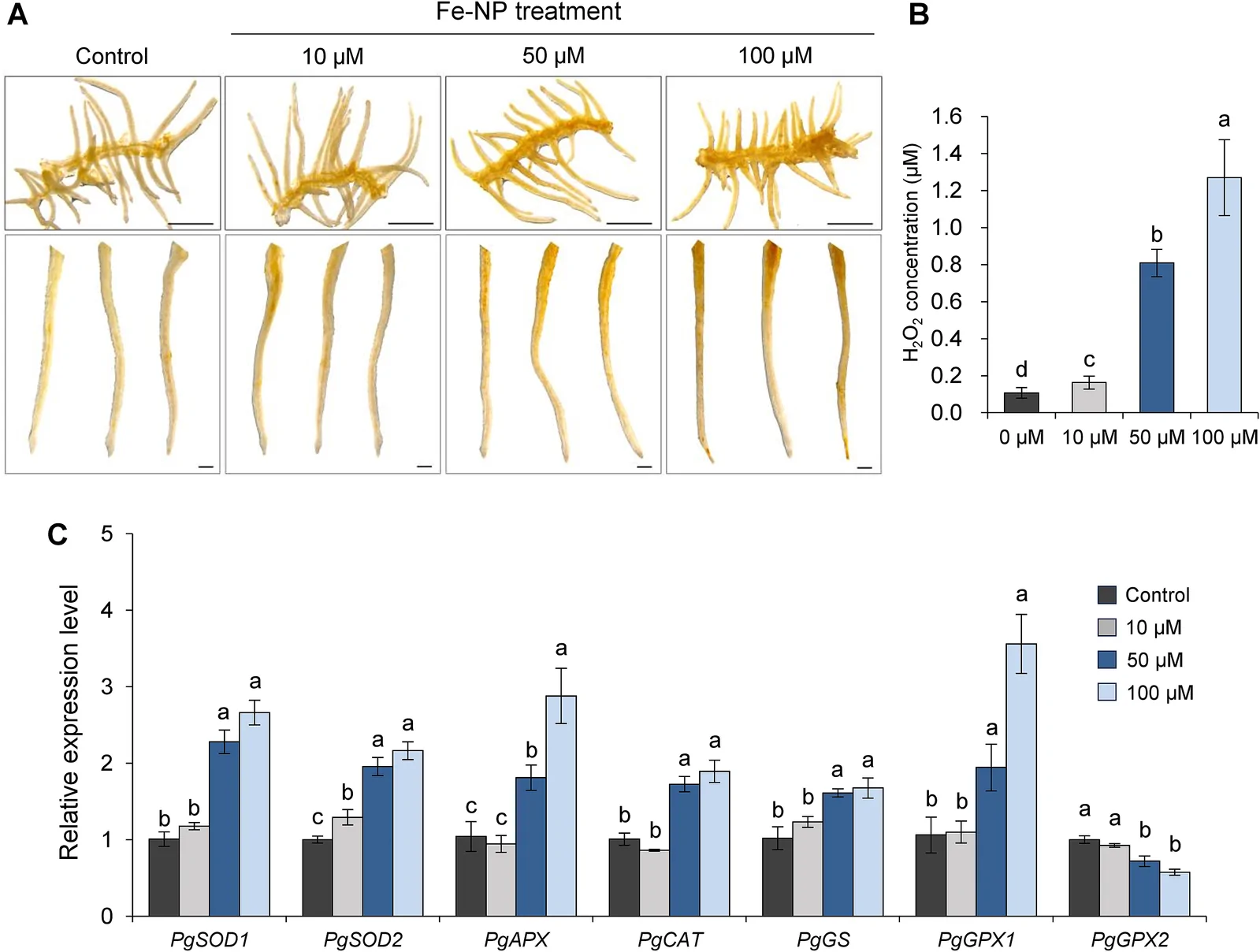
The distribution of ROS in ginseng adventitious roots and ROS metabolism-related gene expression patterns upon different concentrations of Fe-NP. (A) The distribution of ROS in the root tips. ROS were determined by the presence of H_2_O_2_ after the ginseng adventitious roots were treated with Fe-NP for 96 h. Bars: 5 mm for upper images, and 1 mm for lower images. (B) Quantification of H_2_O_2_ in the control and Fe-NP treated ginseng adventitious roots. Data are presented as mean ± SD from four independent replicates. Different superscript letters indicate statistically significant differences (*P < *0.05) determined by one-way ANOVA with Tukey's post hoc test. (C) Expression levels of *PgSOD1, PgSOD2, PgAPX, PgCAT, PgGS, PgGPX1*, and *PgGPX2*. Data represent means ± SD for three independent replicates (n = 3 per group). Different superscript letters indicate statistically significant differences (*P < *0.05) determined by one-way ANOVA with Tukey's *post hoc* test.

To gain mechanistic insight into the increased ROS accumulation in response to Fe-NP treatment, we quantified the relative expression of selected genes involved in ROS metabolism. The expression of two superoxide dismutase genes, *PgSOD1* and *PgSOD2*, which facilitate the conversion of superoxide anions to H_2_O_2_, increased as of Fe-NP concentrations rose from 50 μM to 100 μM (Fig. 5C). Similarly, the transcript levels of H_2_O_2_ scavenging genes, *ascorbate peroxidase* (*PgAPX*), *catalase* (*PgCAT*), *glutathione synthase* (*PgGS*) were significantly elevated at 50 μM and 100 μM Fe-NP (Fig. 5C). In contrast, the two glutathione peroxidase genes, *PgGPX1* and *PgGPX2*, exhibit distinct expression patterns in response to Fe-NP treatment. Notably, *PgGPX1* expression increased by more than 2-fold and 3.5-fold at 50 and 100 μM, respectively (Fig. 5C). In contrast, the *glutathione peroxidase 2* (*PgGPX2*), showed a different expression pattern and did not correlate with the H_2_O_2_ levels, suggesting a lesser role in ROS scavenging (Fig. 5C). Interestingly, the expression of ROS-related genes remained largely unchanged at 10 μM Fe-NP, likely reflecting the moderate ROS levels observed at these concentrations (Fig. 5B and C). At higher Fe-NP expression levels of all examined ROS-related genes increased, except *PgGPX2*. Overall, these results indicate that exposure to high Fe-NP concentrations induces oxidative stress in ginseng adventitious roots, leading to elevated ROS accumulation that may negatively influence ginsenoside biosynthesis.

## 4 Discussion

Iron is an essential micronutrient that plays a pivotal role in plant growth and productivity, and its deficiency disrupts fundamental physiological and biochemical processes [29]. In recent years, the application of nanoparticles (NPs), including iron nanoparticles (Fe-NPs), has attracted considerable attention in agriculture due to their nanoscale size, which enhances bioavailability and cellular uptake [19]. Nanoparticles in the 40–50 nm size range have been shown to be particularly effective for cellular accumulation, thereby improving nutrient delivery efficiency [[30], [31], [32]]. In the present study, Fe-NPs with particles sizes of 20-30 nm were used, a range that facilitates efficient cellular uptake and movement within plant tissues. Fe-NP uptake into ginseng adventitious root tissue was directly confirmed by TEM-EDS (Fig. 1). However, despite these advantages, precise control of Fe-NP concentration is essential to maximize efficacy while minimizing potential risks associated with iron imbalance. Our results demonstrate that optimal Fe-NP concentration has a pronounced effect on ginsenoside biosynthesis in ginseng adventitious roots. Consistent with the essential role of iron in plant physiology, previous studies have demonstrated that iron deficiency significantly reduces biomass and seed yield production in crops such as tomato, spinach, and rice [33]. In addition to its role in primary metabolism, iron availability also influences secondary metabolites production, including phenolics and flavonoid compounds, in lettuce and pepper seedlings [34,35]. Consistent with this, field studies in ginseng have shown that foliar application of conventional iron at optimal concentrations increased biomass by approximately 13% and enhanced total ginsenosides content by 0.7 to 20% [36]. Interestingly, treatment of ginseng adventitious roots with 10 μM of Fe-NP significantly increased the transcript levels of key genes, including *PgDDS, PgOAS, PgPPDS,* and *PgPPTS* (Fig. 2). This treatment increased root biomass by over 32% and maintained total ginsenosides levels 14–15% higher than the control (Figs. 3A and 4, S4). Consistent with this principle, 10 μM Fe-NP markedly enhanced ginsenoside accumulation, whereas an equivalent concentration of conventional Fe_3_O_4_ produced no comparable response (Fig. 3B–Table S3). This contrast demonstrates that biosynthetic stimulation is not attributable to iron itself but to the nanoscale formulation, which likely improves cellular accessibility, uptake dynamics, and intracellular delivery. These findings highlight nanoparticle-specific bioactivity as the critical determinant driving ginsenoside enhancement. The enhanced ginsenoside accumulation may be linked to a moderate elevation in ROS levels (1.6-fold) under 10 μM Fe-NP treatment (Fig. 5A and B), which did not induce oxidative stress, as evidenced by the unchanged expression levels of ROS metabolism-related genes (Fig. 5C), but may instead act as a signaling cue to stimulate ginsenoside biosynthesis. Collectively, these findings suggest that Fe-NP could offer potential advantages over conventional iron fertilizers, likely through improved root uptake efficiency, ultimately enhancing ginsenoside production.

Iron is primarily allocated to chloroplasts, which represents the major sites of iron utilization, accounting for approximately 70 to 90% of total cellular iron in mesophyll cells. In chloroplasts, iron serves as an essential enzyme cofactor, supporting key physiological processes such as photosynthetic electron transfer in protochlorophyll and chlorophyll formation [21,37]. Consistent with this role, Fe-NP application has been shown to significantly increase chlorophyll *a* and *b* contents, thereby enhancing photosynthetic efficiency and promoting overall plant growth [22,38]. However, in non-photosynthetic organs such as ginseng adventitious roots, the physiological role of iron is more limited due to the absence of photosynthetic machinery. Consequently, excess iron may accumulate in root tissues, increasing the risk of iron toxicity and reduced yield. Previous reports have reported that excessive iron supply induces visible toxicity symptoms, including root growth inhibition and biomass reduction [[39], [40], [41]]. Our results indicate that elevated Fe-NP concentrations negatively influence ginseng adventitious roots, reducing ginsenoside accumulation by up to 16% and decreasing biomass production (Fig. 3, Fig. 4, S4). This inhibitory effect likely reflects the dual role of iron in plant physiology: while essential for many biochemical processes, excess iron can promote ROS generation, thereby suppressing plant growth [42,43]. Here, we observed ROS levels in ginseng adventitious roots drastically increased under 50 and 100 μM Fe-NP treatments (Fig. 5A). Consistently, the expression of several ROS-related genes, including *PgSOD1, PgSOD2, PgAPX, PgCAT, PgGS,* and *PgGPX1,* was significantly upregulated at high Fe-NP concentrations (Fig. 5C), indicating that excessive Fe-NP induces iron toxicity and oxidative stress. This response is likely driven by unregulated iron participation in Fenton reactions, which catalyze the conversion of H_2_O_2_ into highly reactive hydroxyl radicals that detrimental to plant health [42,44]. These findings highlight the importance of maintaining iron homeostasis to prevent ROS-mediated toxicity and yield loss.

Oxidative stress resulting from nutrient imbalance is known to disrupt physiological processes and reshape secondary metabolite biosynthesis [45]. In line with this, our results show a shift in ginsenoside composition under high Fe-NP exposure, with PPT-type ginsenosides, represented by Re, accumulating to higher levels than PPD-type compounds (Fig. 3, Fig. 4, S5). We hypothesize that this shift reflects defense-related metabolic adjustment in ginseng adventitious roots aimed at mitigating Fe-NP-induced oxidative stress. Pharmacological studies have shown that Re positively contributed to ROS scavenging by inducing *GPX1* expression, which facilitates H_2_O_2_ detoxification and limits hydroxyl radical formation [46,47]. Furthermore, *PgGPX1* is highly responsive to abiotic stress, particularly H_2_O_2_ exposure, underscoring its role in ROS homeostasis [48,49].

Notably, *PgGPX1* was the most upregulated ROS-scavenging gene under high Fe-NP treatments and showed a positive correlation with the increased proportion of Re ginsenoside (Fig. 5). Although this association suggests a potential link between Re accumulation and enhanced ROS-scavenging capacity, it remains correlative. Further studies are required to determine whether Re plays a directly modulates ROS detoxification pathways, particularly through *PgGPX1*-mediated mechanisms, in ginseng.

## 5 Conclusion

Fe-NPs significantly elevated total ginsenoside content (10 μM), whereas an equivalent dose of conventional Fe_3_O_4_ produced no comparable effect, underscoring the unique bioactivity of the nanoparticle formulation. In contrast, higher concentrations (50-100 μM) triggered oxidative stress, leading to ROS accumulation and a subsequent decline in ginsenoside production (Fig. 6). This stress-driven shift was accompanied by metabolic reprogramming, characterized by reduced PPD-type ginsenosides and increased PPT-type Re, likely mediated by ROS-responsive pathways such as *PgGPX1*. These findings highlight the importance of precise Fe-NP dosage control to maximize elicitation efficiency while minimizing oxidative stress. Overall, this work provides a mechanistic framework for understanding Fe-NP–mediated regulation of ginsenoside biosynthesis and establishes a foundation for exploring nanoparticle-based enhancement of secondary metabolism in medicinal plants.

**Fig. 6:**
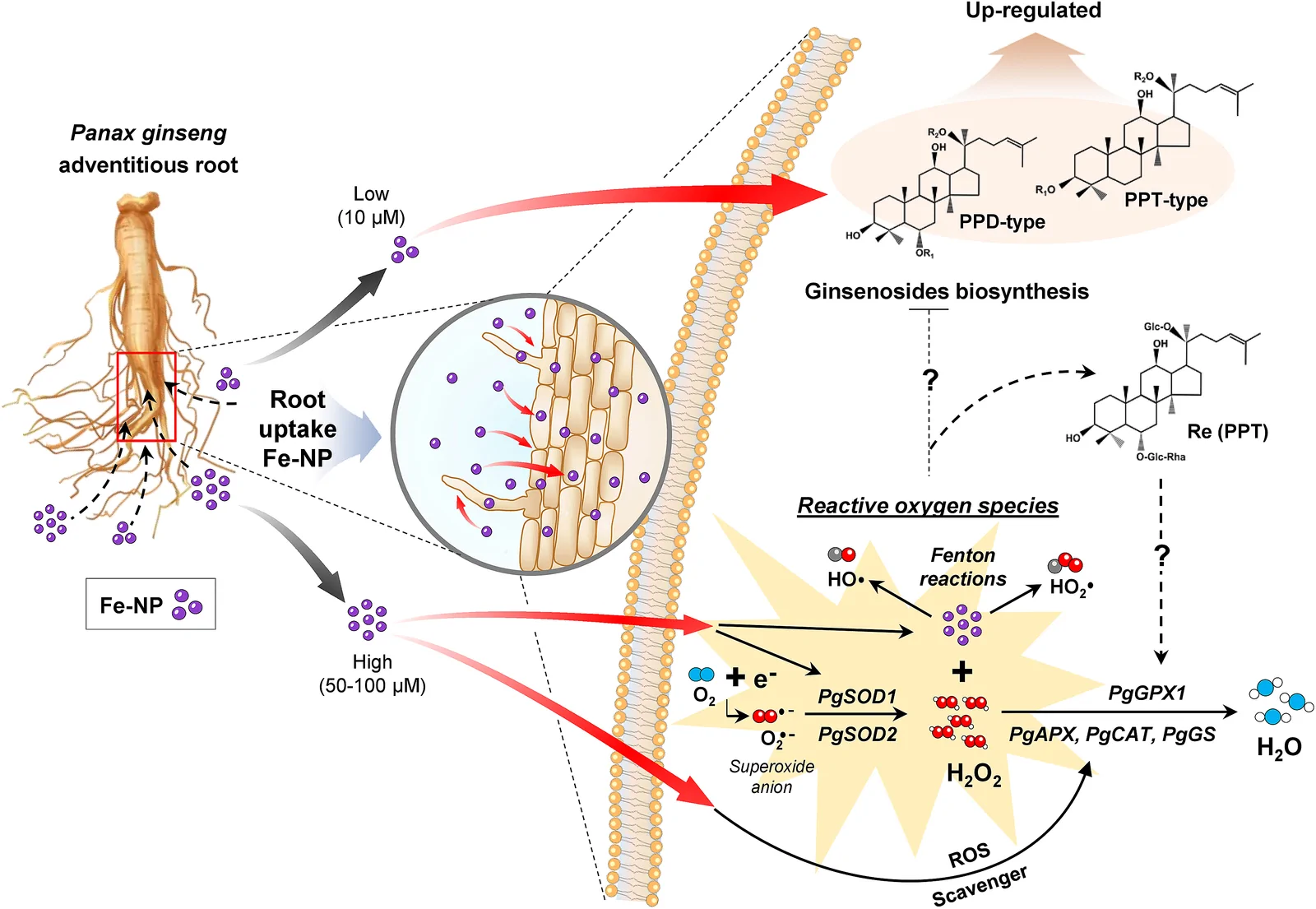
A schematic illustration of ginseng adventitious roots exhibits distinct responses to various Fe-NP concentrations. At 10 μM, Fe-NP treatment significantly enhances ginsenoside production. However, at higher concentrations, Fe-NP triggers the production of reactive oxygen species (ROS), which inhibits ginsenoside biosynthesis. Additionally, the content of Re ginsenoside increases at higher Fe-NP levels, potentially contributing to the ROS scavenging mechanism. Straight lines mean direct interactions, while dashed lines indicate hypothetical or indirect interactions.

## Authorship contributions

O.R.L conceptualized the idea. U.B., J.H.J., and O.R.L conducted the experiments and analyzed the data. U.B., J.H.J, S.P.K, and O.R.L., wrote the paper.

## Data availability and statements

Experimental data generated during this study are fully available in the manuscript and its Supplementary Materials.

## Declaration of competing interest

No potential conflicts of interest are declared by the authors in relation to this manuscript.

## Figures and Tables

**Fig. S1:**
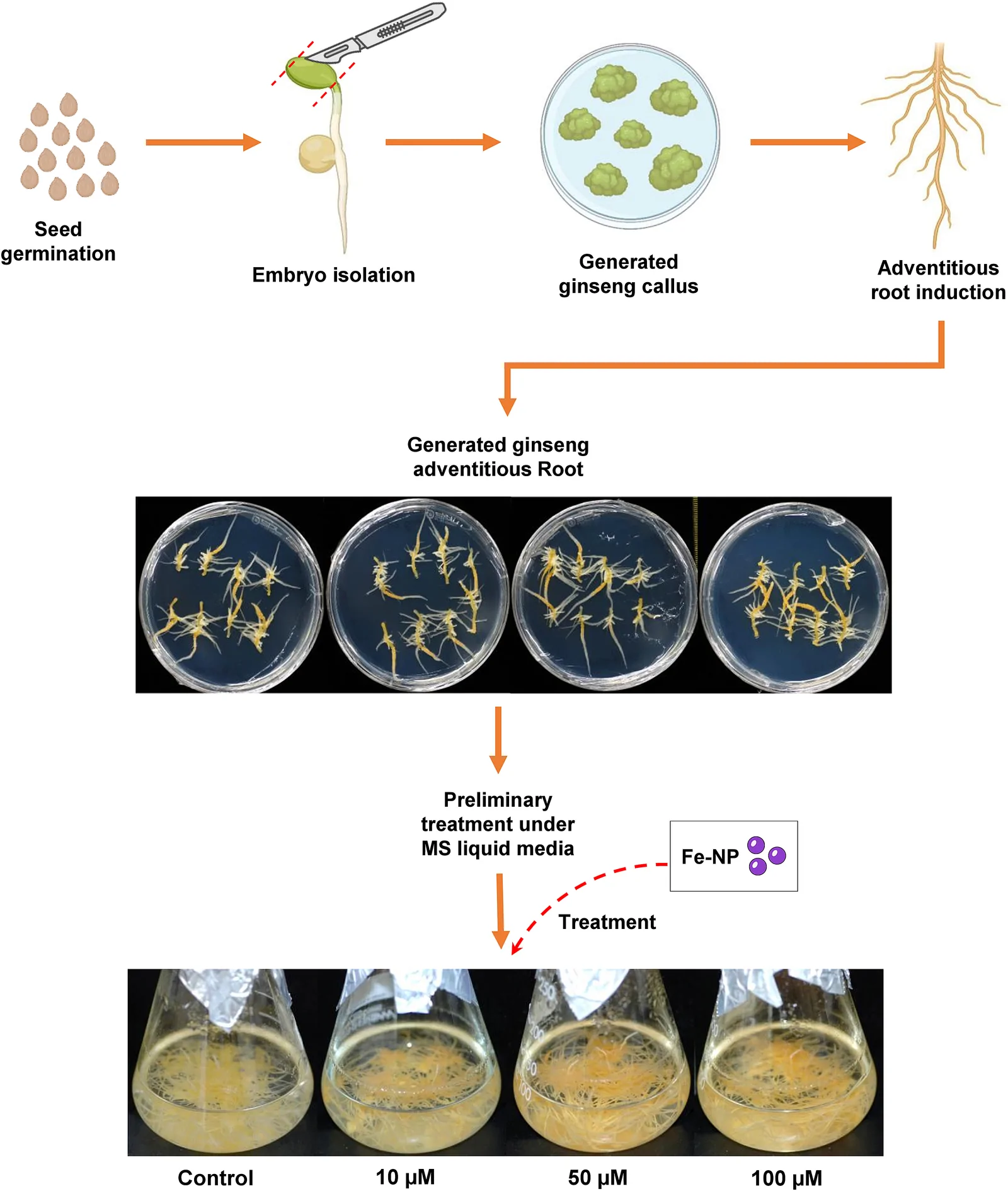
A schematic diagram of the experimental steps for Fe-NP treatment in ginseng adventitious roots. Initially, ginseng callus was generated from embryos isolated from seedlings. This callus was then induced to develop into adventitious roots, which served as the material for testing the effects of Fe-NP treatment.

**Fig. S2:**
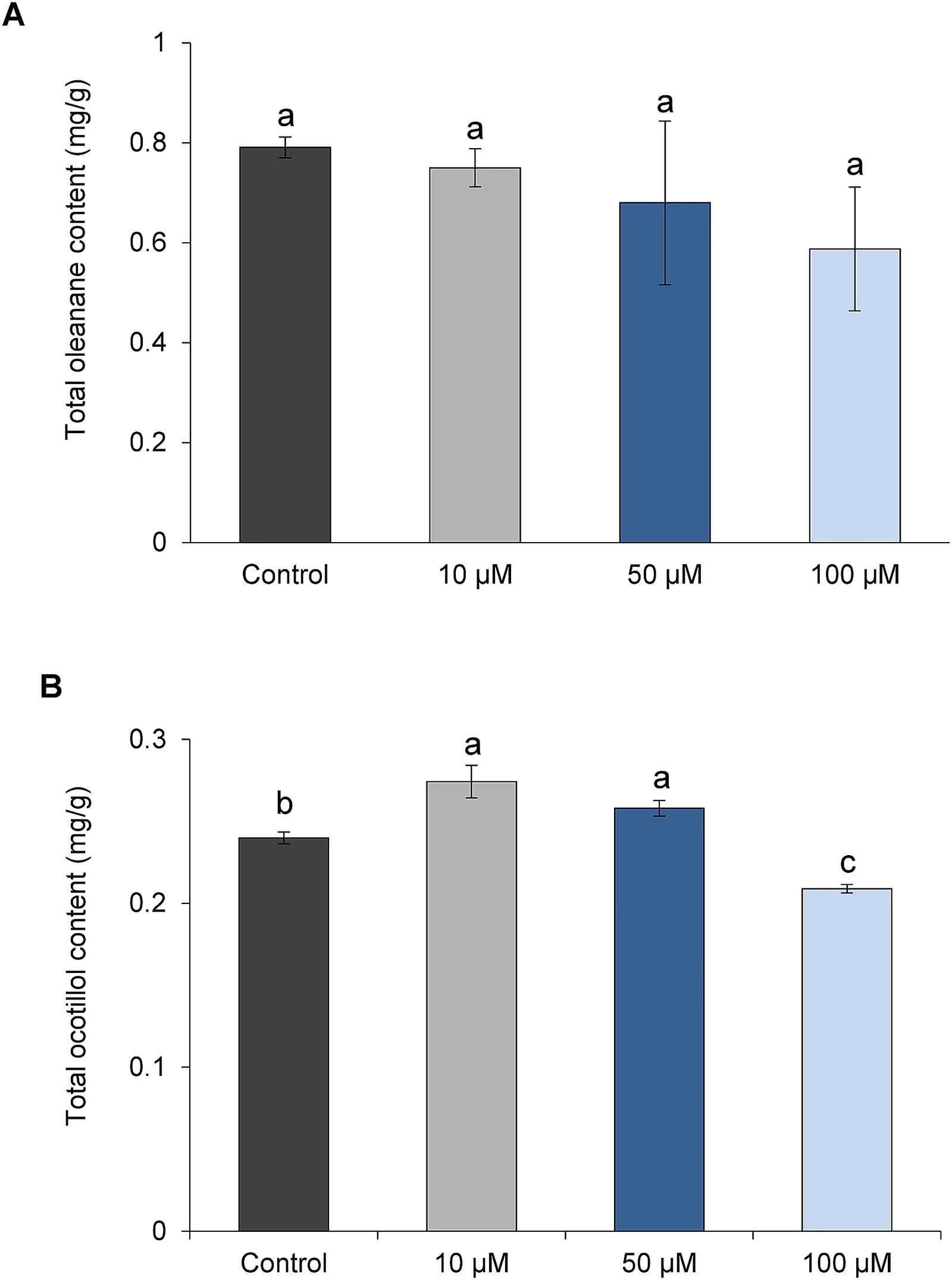
Profiles of oleanane and ocotillol ginsenosides. (A & B) Oleanane-type (Ro) and ocotillol-type (PF11) ginsenosides detected in ginseng adventitious roots. Data are presented as mean ± SD from three independent biological replicates (n = 3 per group). Statistical significance was assessed using one-way ANOVA followed by Tukey's *post hoc* test; different superscript letters indicate significant differences (*P < *0.05).

**Fig. S3:**
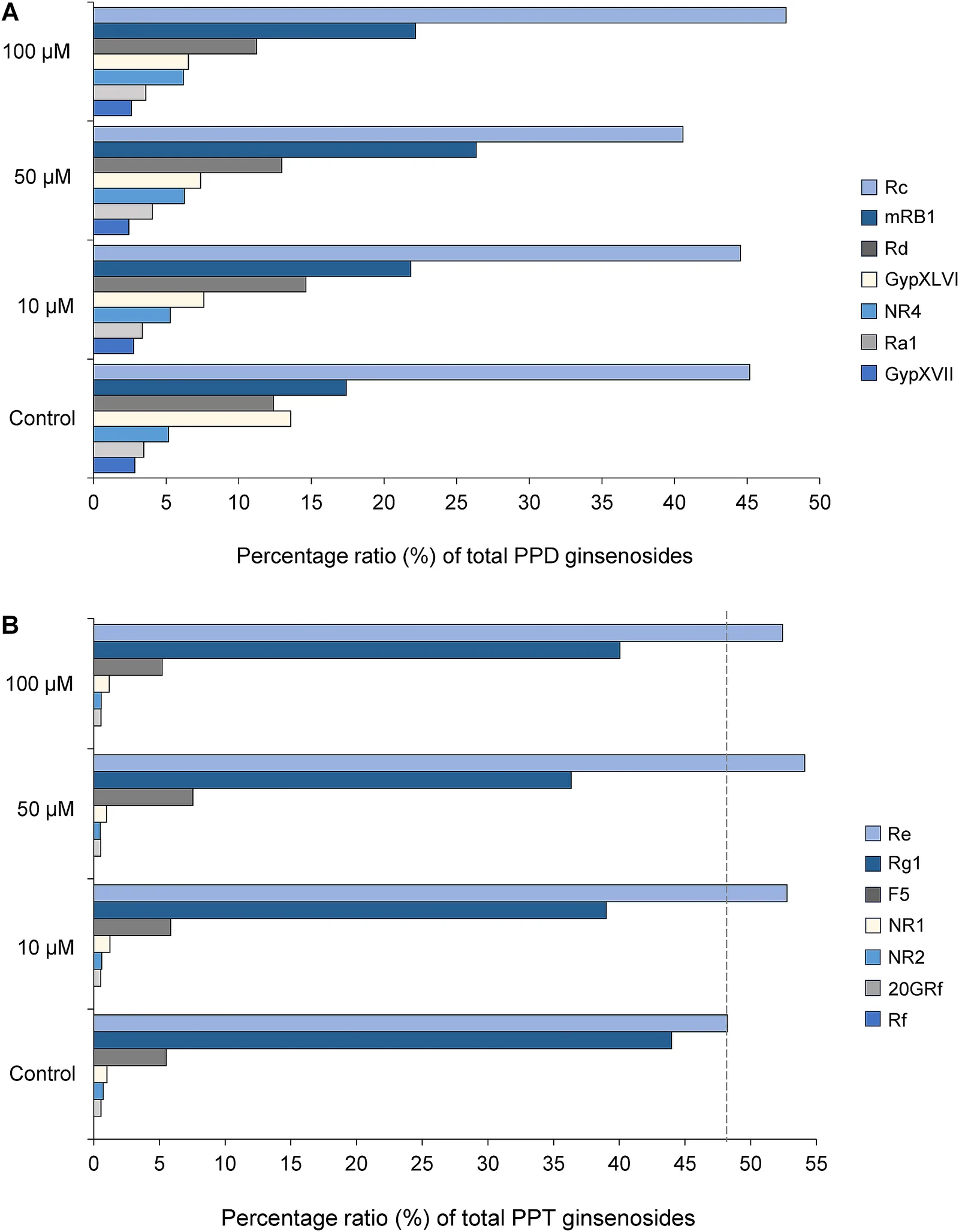
Percentage ratio of the individual PPD and PPT ginsenosides under different concentrations of Fe-NP. (A & B) Ratio of individual PPD- and PPT-type ginsenosides detected in ginseng adventitious roots after Fe-NP treatment. The percentage ratio was calculated based on the total content of PPD- or PPT-type ginsenosides at each Fe-NP concentration. Data are presented as mean ± SD from three independent biological replicates (n = 3 per group).

**Fig. S4:**
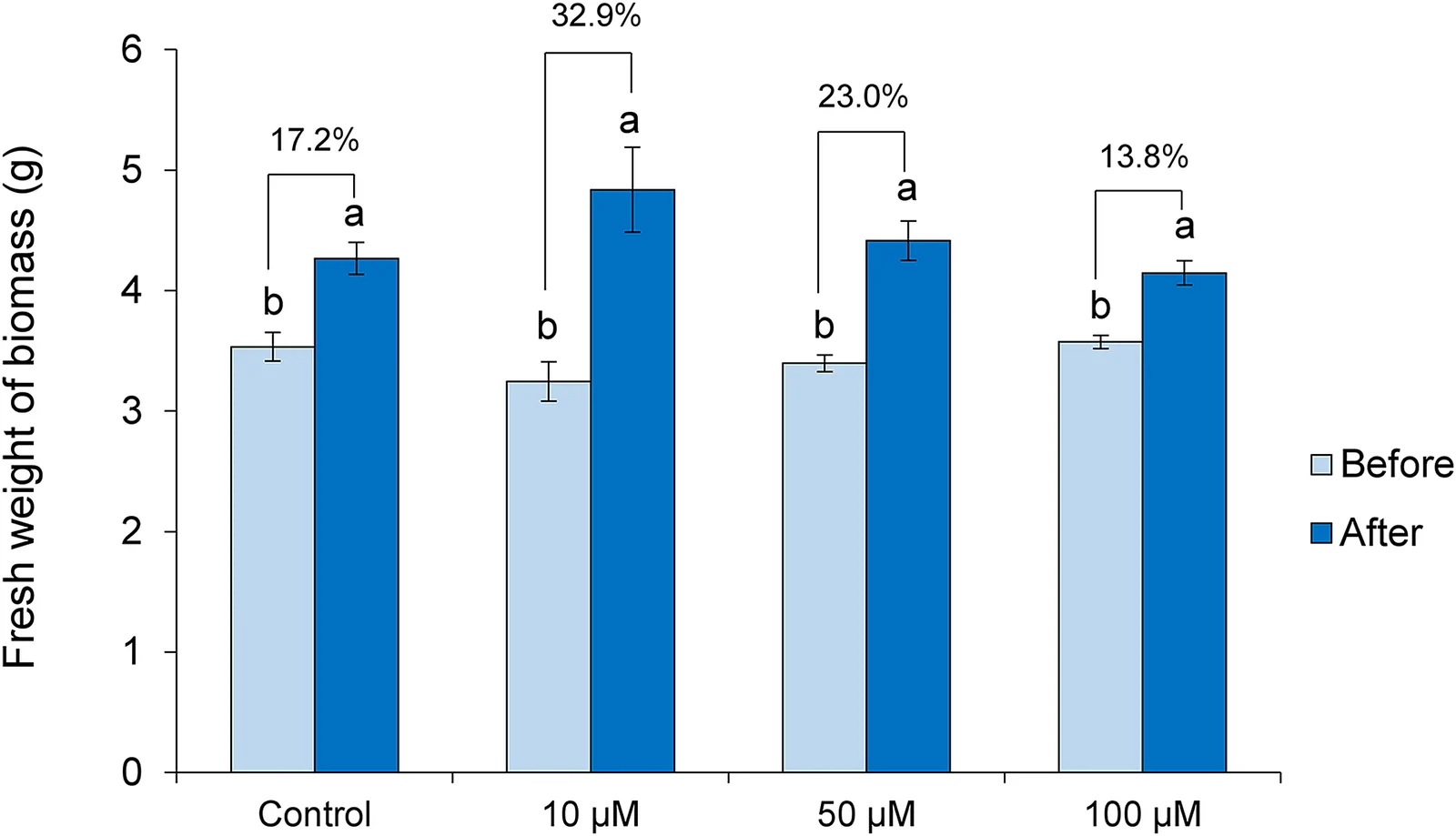
The biomass content in ginseng adventitious roots under different concentrations of Fe-NP. Fresh weight of adventitious root biomass before and after treatment. All data are expressed as the mean ± SD from three independent biological replicates (n = 3 per group). Statistical significance was determined by one-way ANOVA with Tukey's *post hoc* test; superscript letters indicate significant differences (*P* < 0.05). The number on the top of each bar graph indicates the percentage increase of fresh weight after treatment.

**Fig. S5:**
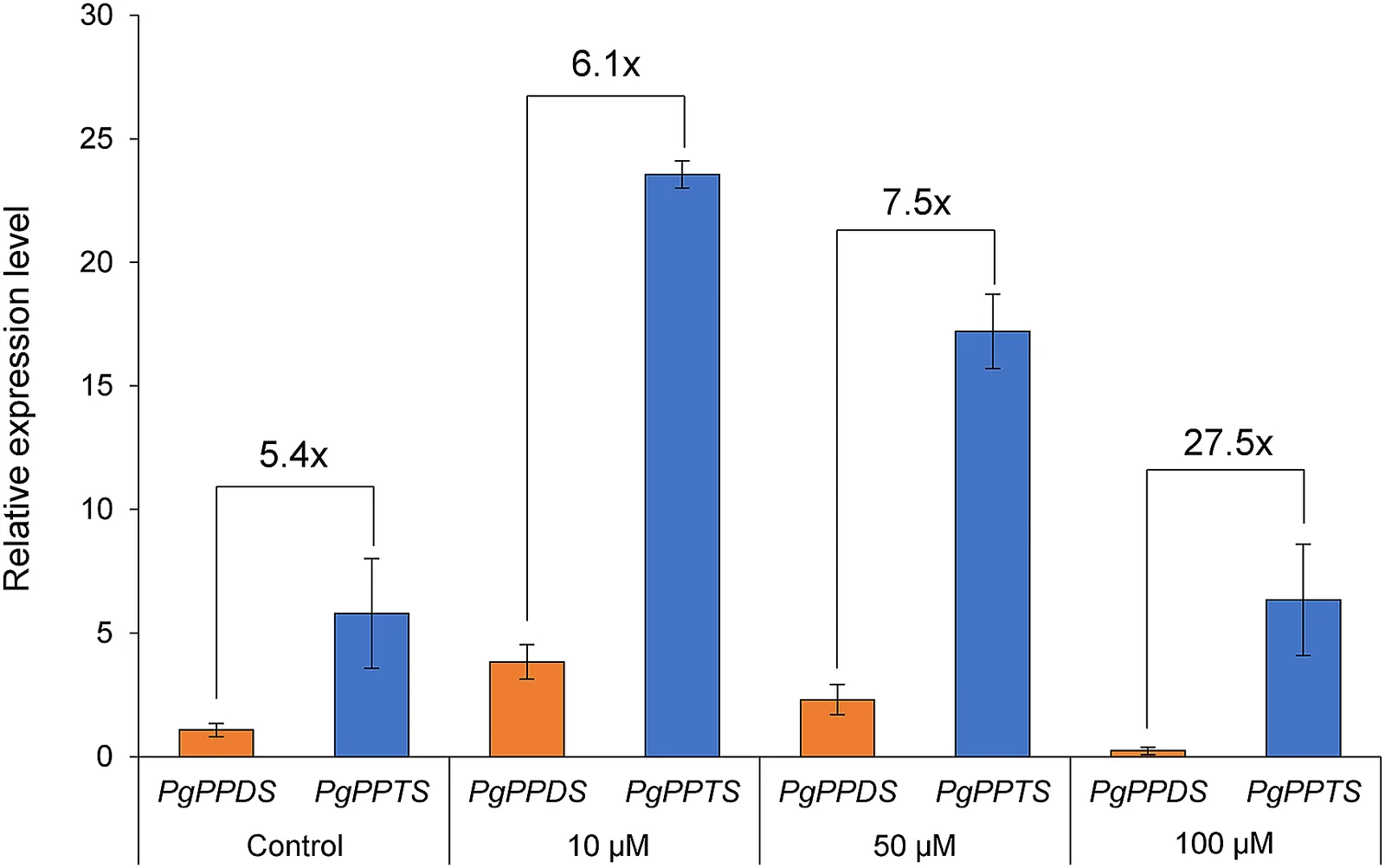
Expression level of the *PPDS* gene compared to the *PPTS* gene upon Fe-NP treatment. All data are expressed as the mean ± SD from three independent biological replicates (n = 3 per group). Statistical significance was determined by one-way ANOVA with Tukey's *post hoc* test; superscript letters indicate significant differences (*P* < 0.05).

## Appendix A Supplementary data

Table S1. List of primers used for gene expression analysis in this study.Multimedia component. 1

Table S2 and S3. Quantification of ginsenoside contents under different concentrations of Fe-NP and conventional iron (Fe_3_O_4_).Multimedia component. 2

## References

[1] Kim Y.J., Zhang D., Yang D.C. (2015). Biosynthesis and biotechnological production of ginsenosides. Biotechnol Adv.

[2] Kim Y.J., Lee O.R., Oh J.Y., Jang M.G., Yang D.C. (2014). Functional analysis of 3-hydroxy-3-methylglutaryl coenzyme A reductase encoding genes in triterpene saponin-producing ginseng. Plant Physiol.

[3] Hou M., Wang R., Zhao S., Wang Z. (2021). Ginsenosides in *Panax* genus and their biosynthesis. Acta Pharm Sin B.

[4] Han J.Y., In J.G., Kwon Y.S., Choi Y.E. (2010). Regulation of ginsenoside and phytosterol biosynthesis by RNA interferences of squalene epoxidase gene in *Panax ginseng*. Phytochemistry.

[5] Han J.Y., Jo H.J., Choi Y.E. (2020). Overexpression of the squalene epoxidase gene (*PgSE1*) resulted in enhanced production of ginsenosides and phytosterols in transgenic ginseng. Plant Biotechnol Rep.

[6] Han J.Y., Kwon Y.S., Yang D.C., Jung Y.R., Choi Y.E. (2006). Expression and RNA interference-induced silencing of the dammarenediol synthase gene in *Panax ginseng*. Plant Cell Physiol.

[7] Baeg I.-H. (2022). The global ginseng market and Korean ginseng. J Ginseng Cult.

[8] Chopra P., Chhillar H., Kim Y.J., Jo I.H., Kim S.T., Gupta R. (2023). Phytochemistry of ginsenosides: recent advancements and emerging roles. Crit Rev Food Sci Nutr.

[9] Chu L.L., Montecillo J.A.V., Bae H. (2020). Recent advances in the metabolic engineering of yeasts for ginsenoside biosynthesis. Front Bioeng Biotechnol.

[10] Dai Z., Wang B., Liu Y., Shi M., Wang D., Zhang X. (2014). Producing aglycons of ginsenosides in bakers' yeast. Sci Rep.

[11] Sangwan N.S., Jha S., Mitra A. (2024). Unlocking nature's treasure trove: biosynthesis and elicitation of secondary metabolites from plants. Plant Growth Regul.

[12] Zhang W., Yu P., Liu W., Wang L., Song X., Yao Y. (2025). Mechanism of sodium nitroprusside regulating ginseng quality. Sci Rep.

[13] Dey A., Sadhukhan A. (2024 1146 2024). Molecular mechanisms of plant productivity enhancement by nano fertilizers for sustainable agriculture. Plant Mol Biol.

[14] Ulusoy E., Bozkurt A., Durmaz S., Servi H., Vardar F., Erisen S. (2024). Impact of silver nanoparticles on secondary metabolite composition and toxicity in anise (*Pimpinella anisum* L.) callus culture. BMC Plant Biol.

[15] Mazaheri-Tirani M., Kashani A., Koohi-Dehkordi M. (2022). The role of iron nanoparticles on morpho-physiological traits and genes expression (IRT1 and CAT) in rue (*Ruta graveolens*). Plant Mol Biol.

[16] Andrés-Bordería A., Andrés F., Garcia-Molina A., Perea-García A., Domingo C., Puig S. (2017). Copper and ectopic expression of the arabidopsis transport protein COPT1 alter iron homeostasis in rice (*Oryza sativa* L.). Plant Mol Biol.

[17] Schmidt S.B., Jensen P.E., Husted S. (2016). Manganese deficiency in plants: the impact on photosystem II. Trends Plant Sci.

[18] Humbal A., Pathak B. (2023). Influence of exogenous elicitors on the production of secondary metabolite in plants: a review (“VSI: secondary metabolites”. Plant Stress.

[19] Selvakesavan R.K., Kruszka D., Shakya P., Mondal D., Franklin G. (2023). Nanomaterial interactions with plant cellular mechanisms and macromolecules and agricultural implications.

[20] Singh Y., Kumar U., Panigrahi S., Balyan P., Mehla S., Sihag P. (2023). Nanoparticles as novel elicitors in plant tissue culture applications: current status and future outlook. Plant Physiol Biochem.

[21] Ning X., Lin M., Huang G., Mao J., Gao Z., Wang X. (2023). Research progress on iron absorption, transport, and molecular regulation strategy in plants. Front Plant Sci.

[22] Li P., Xia Y., Song K., Liu D. (2024). The impact of nanomaterials on photosynthesis and antioxidant mechanisms in gramineae plants: research progress and future prospects. Plants.

[23] Kokina I., Plaksenkova I., Galek R., Jermaļonoka M., Kirilova E., Gerbreders V. (2021). Genotoxic evaluation of Fe3O4 nanoparticles in different three barley (*Hordeum vulgare* L.) genotypes to explore the stress-resistant molecules. Molecules.

[24] Rezayian M., Niknam V., Arabloo M. (2023). Iron nanoparticle regulate succinate dehydrogenase activity in canola plants under drought stress. Sci Rep.

[25] Razavizadeh R., Anwari A. al S., Forghani A.H., Mirmazloum I. (2024). Application of iron oxide nanoparticles improves growth and phytochemical constituents of *in vitro* cultured *Carum copticum* L. J Agric Food Res.

[26] Lee O.R., Han J.H., Kim Y.-R. (2014). Agrobacterium-mediated transformation of mature ginseng embryos. Bio-protocol.

[27] Hao M., Tan X., Liu K., Xin N. (2025). Nanoencapsulation of nutraceuticals: enhancing stability and bioavailability in functional foods. Front Nutr.

[28] Souza L.R.R., Bernardes L.E., Barbetta M.F.S., da Veiga M.A.M.S. (2019). Iron oxide nanoparticle phytotoxicity to the aquatic plant lemna minor: effect on reactive oxygen species (ROS) production and chlorophyll a/chlorophyll b ratio. Environ Sci Pollut Res.

[29] Morrissey J., Lou Guerinot M. (2009). Iron uptake and transport in plants: the good, the bad, and the ionome. Chem Rev.

[30] Sun X.D., Yuan X.Z., Jia Y., Feng L.J., Zhu F.P., Dong S.S. (2020). Differentially charged nanoplastics demonstrate distinct accumulation in *Arabidopsis thaliana*. Nat Nanotechnol.

[31] Sun X.D., Ma J.Y., Feng L.J., Duan J.L., Xie X.M., Zhang X.H. (2023). Magnetite nanoparticle coating chemistry regulates root uptake pathways and iron chlorosis in plants. Proc Natl Acad Sci U S A.

[32] Sun X.D., Ma J.Y., Feng L.J., Duan J.L., Yuan X.Z. (2024). Precise tracking of nanoparticles in plant roots. Nat Protoc.

[33] Briat J.F., Dubos C., Gaymard F. (2015). Iron nutrition, biomass production, and plant product quality. Trends Plant Sci.

[34] Kalisz A., Húska D., Jurkow R., Dvořák M., Klejdus B., Caruso G. (2021). Nanoparticles of cerium, iron, and silicon oxides change the metabolism of phenols and flavonoids in butterhead lettuce and sweet pepper seedlings. Environ Sci Nano.

[35] Ahamed T.E.S., Ahamed E.S.S. (2018). Synergy prospect low gamma irradiation doses incorporating elicitation with iron nanoparticles to hyper production biomass yield and bioactive secondary metabolites for cress, medicinal plant. J Plant Sci.

[36] Zhang H., Yang H., Wang Y., Gao Y., Zhang L. (2013). The response of ginseng grown on farmland to foliar-applied iron, zinc, manganese and copper. Ind Crops Prod.

[37] Marschner H., Römheld V. (1994). Strategies of plants for acquisition of iron. Plant Soil.

[38] Feng Y., Kreslavski V.D., Shmarev A.N., Ivanov A.A., Zharmukhamedov S.K., Kosobryukhov A. (2022). Effects of iron oxide nanoparticles (Fe3O4) on growth, photosynthesis, antioxidant activity and distribution of mineral elements in wheat (*Triticum aestivum*) plants. Plants.

[39] Zhang L., Li G., Wang M., Di D., Sun L., Kronzucker H.J. (2018). Excess iron stress reduces root tip zone growth through nitric oxide-mediated repression of potassium homeostasis in arabidopsis. New Phytol.

[40] Sonu Nandakumar S., Singh V.J., Pandey R., Gopala Krishnan S., Bhowmick P.K. (2023). Implications of tolerance to iron toxicity on root system architecture changes in rice (*Oryza sativa* L.). Front Sustain Food Syst.

[41] Chatterjee C., Gopal R., Dube B.K. (2006). Impact of iron stress on biomass, yield, metabolism and quality of potato (*Solanum tuberosum* L.). Sci Hortic (Amsterdam).

[42] Wu L.B., Ueda Y., Lai S.K., Frei M. (2017). Shoot tolerance mechanisms to iron toxicity in rice (*Oryza sativa* L.). Plant Cell Environ.

[43] Reyt G., Boudouf S., Boucherez J., Gaymard F., Briat J.F. (2015). Iron- and ferritin-dependent reactive oxygen species distribution: impact on arabidopsis root system architecture. Mol Plant.

[44] Winterbourn C.C. (1995). Toxicity of iron and hydrogen peroxide: the fenton reaction. Toxicol Lett.

[45] Panahirad S., Morshedloo M.R., Ali S., Hano C., Kulak M. (2023). Secondary metabolites and their potential roles in plant tolerance against abiotic and biotic stress. Plant Stress.

[46] Shin E.J., Nguyen B.T., Sharma N., Tran N.K.C., Nguyen Y.N.D., Hwang Y. (2023). Ginsenoside re mitigates memory impairments in aged GPx-1 KO mice by inhibiting the interplay between PAFR, NFκB, and microgliosis in the hippocampus. Food Chem Toxicol.

[47] Liu M., Bai X., Yu S., Zhao W., Qiao J., Liu Y. (2019). Ginsenoside re inhibits ROS/ASK-1 dependent mitochondrial apoptosis pathway and activation of Nrf2-Antioxidant response in beta-amyloid-challenged SH-SY5Y cells. Molecules.

[48] Kim Y.J., Jang M.G., Noh H.Y., Lee H.J., Sukweenadhi J., Kim J.H. (2014). Molecular characterization of two glutathione peroxidase genes of *Panax ginseng* and their expression analysis against environmental stresses. Gene.

[49] Sathiyaraj G., Lee O.R., Parvin S., Khorolragchaa A., Kim Y.J., Yang D.C. (2011). Transcript profiling of antioxidant genes during biotic and abiotic stresses in *Panax ginseng*. Mol Biol Rep.

